# Genomic Information for Clinicians in the Electronic Health Record: Lessons Learned From the Clinical Genome Resource Project and the Electronic Medical Records and Genomics Network

**DOI:** 10.3389/fgene.2019.01059

**Published:** 2019-10-29

**Authors:** Marc S. Williams, Casey Overby Taylor, Nephi A. Walton, Scott R. Goehringer, Samuel Aronson, Robert R. Freimuth, Luke V. Rasmussen, Eric S. Hall, Cynthia A. Prows, Wendy K. Chung, Alexander Fedotov, Jordan Nestor, Chunhua Weng, Robb K. Rowley, Georgia L. Wiesner, Gail P. Jarvik, Guilherme Del Fiol

**Affiliations:** ^1^Genomic Medicine Institute, Geisinger, Danville, PA, United States; ^2^Department of Medicine, Johns Hopkins University, Baltimore, MD, United States; ^3^Partners HealthCare, Boston, MA, United States; ^4^Department of Health Sciences Research, Mayo Clinic, Rochester, MN, United States; ^5^Department of Preventive Medicine, Northwestern University, Chicago, IL, United States; ^6^Department of Pediatrics, University of Cincinnati College of Medicine, and Cincinnati Children’s Hospital Medical Center, Cincinnati, OH, United States; ^7^Divisions of Human Genetics and Patient Services, Cincinnati Children’s Hospital Medical Center, Cincinnati, OH, United States; ^8^Departments of Pediatrics and Medicine, Columbia University, New York, NY, United States; ^9^Irving Institute for Clinical and Translational Research, Columbia University, New York, NY, United States; ^10^Department of Medicine, Division of Nephrology, Columbia University, New York, NY, United States; ^11^Department of Biomedical Informatics, Columbia University, New York, NY, United States; ^12^National Human Genome Research Institute, Bethesda, MD, United States; ^13^Division of Genetic Medicine, Department of Internal Medicine, Vanderbilt University Medical Center, Nashville, TN, United States; ^14^Departments of Medicine (Medical Genetics) and Genome Sciences, University of Washington, Seattle, WA, United States; ^15^Department of Biomedical Informatics, University of Utah, Salt Lake City, UT, United States

**Keywords:** genomics, electronic health record, education, clinical decision support, infobutton, knowledge synthesis, interoperability, implementation

## Abstract

Genomic knowledge is being translated into clinical care. To fully realize the value, it is critical to place credible information in the hands of clinicians in time to support clinical decision making. The electronic health record is an essential component of clinician workflow. Utilizing the electronic health record to present information to support the use of genomic medicine in clinical care to improve outcomes represents a tremendous opportunity. However, there are numerous barriers that prevent the effective use of the electronic health record for this purpose. The electronic health record working groups of the Electronic Medical Records and Genomics (eMERGE) Network and the Clinical Genome Resource (ClinGen) project, along with other groups, have been defining these barriers, to allow the development of solutions that can be tested using implementation pilots. In this paper, we present “lessons learned” from these efforts to inform future efforts leading to the development of effective and sustainable solutions that will support the realization of genomic medicine.

## Introduction

Genomic information is increasingly used in clinical care. However, genomics can only improve healthcare if clinicians and patients are able to identify when genomic information may be useful and, given the durable nature of genomic information, coupled with increased knowledge that enhances interpretation over time, apply the information over the patient’s life span. Clinicians without genetic training consistently state they are unprepared to use genomic information to care for their patients (Mikat-Stevens et al., 2015; Pet et al., 2019). There is also concern about where to find reliable information to guide the use of genomic results. Traditional educational approaches to improve genomic knowledge are necessary but insufficient, given the dynamic nature of genomic discovery and rapidly changing knowledge relevant to the use of genomics in the care of patients. This necessitates innovative approaches to storage, knowledge synthesis, representation, retrieval, and presentation, ideally integrated into a redesigned clinician workflow supporting the delivery of relevant genomic information provided “just in time” to support clinical care. The electronic health record (EHR) ecosystem is expected to play a key role in this area (Hoffman 2007; Hoffman and Williams, 2011). In this paper, we will review the lessons learned from two large projects developing approaches to educate clinicians within the EHR.

## Materials and Methods

### Setting

The work was done in two large research projects funded by the National Human Genome Research Institute (NHGRI).

The Electronic Medical Records and Genomics (eMERGE) Network^1^ was initially funded in 2007 with the goal of developing and studying the EHR as a tool for genomic research. It is currently completing its third cycle of funding. Phase 1 was a proof of concept that demonstrated that EHR data can be used to develop reliable clinical phenotypes, which can subsequently be used for genomic discovery (primarily for genome-wide association studies). In Phase 2, in addition to expanding the phenotyping work of Phase 1, the network began to explore how the EHR could be used to deliver genomic results to clinicians and patients *via* pilot implementations. Phase 3 has been focused on the implementation of genomic medicine in the clinic, where 25,000 participants were sequenced using targeted next-generation sequencing (eMERGEseq)^2^. This custom assay sequenced a set of 109 actionable genes as well as other single nucleotide variants (SNVs), including genes from version 1 of the American College of Medical Genetics and Genomics (ACMG) secondary findings list (Green et al., 2013). Sites received results to return to participants (Kullo et al., 2014; Jarvik et al., 2014; eMERGE Consortium, 2019).

The Clinical Genome Resource (ClinGen) project was initially funded in 2013. The goal of this project is to increase the medical community’s knowledge about the relationship between genes and health. The primary task is building a knowledge base that defines the clinical relevance of genes and variants for use in precision medicine and research.

Recognizing the importance of the EHR to support the return of results, the Electronic Health Record Integration (EHRI) Working Group was established in Phase 2 of the eMERGE project^3^. The EHRI studied use of the EHR to store genomic test reports and present the results to clinicians and patients. The EHR is also being used to capture patient outcomes related to the return of results. Several tools have been developed by the EHRI that have the potential to impact clinician education. ClinGen established an Electronic Health Record Working Group (EHR WG) tasked with identifying strategies to provide access to ClinGen through the EHR. Liaisons were established between the eMERGE EHRI and ClinGen EHR WG committees to coordinate efforts and accelerate progress. Through their respective evaluation of EHR functionality, the groups developed strategies to accomplish these goals.

Given the novel nature of the problems and resulting strategies, little prior work was available to guide the groups’ respective efforts. Therefore, an exploratory approach was used where potential solutions to problems were developed through an informal group process. Volunteers then tested the prototype solutions in development environments associated with the EHR. The results of these pilot implementations are brought back to the groups for discussion and iterative improvement of the tools. This process, while informal, is informed by conceptual frameworks, or desiderata, for genomic data and clinician education and decision support proposed by Masys et al. (2012) and Welch et al. (2014).

While this paper focuses on two specific initiatives, the NHGRI has other funded projects that are using the EHR for genomic medicine. Liaisons to the relevant workgroups and projects are in place to coordinate efforts and disseminate successful strategies. These will be discussed below.

## Results

In 2012 Masys et al., defined a set of technical desiderata for the integration of genomic data into the EHR (Masys et al., 2012). Analysis of these desiderata by the EHRI and EHR WG has identified numerous barriers that impact the ability to represent ClinGen and eMERGE information in the EHR environment. All of the identified barriers will impact the ability to fully use genomic information as a part of healthcare, and as such, no formal prioritization of impact was performed. There are certain dependencies that exist which were the subject of discussion to fully understand the relationships between the barriers. There was also recognition that some barriers could be overcome using existing platforms and resources to develop local solutions to inform more generalizable approaches, while other barriers would require changes to EHR systems, or international standards that were outside of the direct control of the working groups, although information obtained through trial implementation could be shared with these external entities to inform their development. The information that follows represents a qualitative but pragmatic synthesis of the barriers and potential solutions.

### Standards

Arguably the most important and foundational barrier encountered is the limited ability to transmit gene and variant information as standards-compliant, structured data. This is due to several limitations including: inadequate standards for representing core genomic information, such as gene and variant names and variant classification; lack of standards surrounding the naming and delineation of genetic disease; limited interfaces to access EHR data and external information; suboptimal user experience accessing external resources within the EHR; and lack of input from geneticists, clinicians, and informaticians into vendor design to develop improvements. These limitations have a downstream impact on the ability to provide clinician education within the EHR environment through clinical decision support (CDS) capabilities, including access to point-of-care, just-in-time information relevant for the care of the patient and the ability to integrate this information and associated knowledge within other clinical applications that are critical to clinician workflow. In light of these limitations, some incremental progress towards the goal has been achieved. One example is through the use of a standards-based CDS capability available in the EHR, generally known as “infobuttons” (Del Fiol et al., 2012). Ancillary genomic systems that augment EHR functionality have also been used to provide needed functionality. Improvements in both EHR and ancillary genomic systems, combined with more robust data interfaces such as Fast Healthcare Interoperability Resources (FHIR) (Alterovitz et al., 2015), are providing opportunities for new approaches. These issues are summarized in **Table 1**, and each will be discussed in detail below (Herr et al., 2015; Tenenbaum et al., 2016).

**Table 1:** Requirements, Available Standards, Challenges, and Resources to Support Clinician Education in the Electronic Health Record.

Requirements for clinical genomics implementation	Related standards and resources	Challenges	eMERGE/ClinGen efforts to overcome challenges
Storage of genomic data	Ancillary genomic systems Variant Call Format (VCF)	Inadequate ability of current EHRs to store detailed discrete genomic results Lack of consistent open source reference data structure that can robustly represent results Need to represent heterogeneous result types (e.g., star alleles, diplotypes)	eMERGE XML provides an example of the content such standards should represent
Representation and exchange of patient genomic data in the EHR	HL7 v2 Clinical Genomic Implementation Guide HL7 FHIR Genomic Reporting Implementation Guide GA4GH Variant Representation Specificatione MERGE XML standard	Rapid evolution of data types and use cases related to clinical genomics Slow evolution of HL7 standards Low adoption of extant standards by EHR vendors and genetic testing laboratories	Interviews led by EHRI workgroup with eMERGE and CSER sites to understand intended use of genomic test reports and requirements for transferring reports and associated data from laboratories to sites Development of an XML standard capable of transmitting results within the eMERGE Network Interactions with HL7 to assist in incorporating the eMERGE XML standard into the FHIR standard
Representation and exchange of variant knowledge	ClinGen resource GA4GH Variant Annotation model (in progress) eMERGE XML standard Monarch initiative (for ontology support)	Lack of resources with clinical genomics knowledge in computable format	eMERGE XML development and validation ClinGen resource: Variant Curation Working Groups ClinGen resource: Allele Registry
Clinical decision support (CDS)	HL7 Infobutton Standard, OpenInfobutton SMART on FHIR CDS Hooks standard	Lack of EHR and laboratory support for representation of genetic data in standard formats Lack of clinical genomic resources with knowledge accessible in computable, standards-compliant format Little experience with CDS for the use of genomic data in clinical care Lack of expert guidelines for clinical management of genomic findings to serve as the decision logic for CDS tools	OpenInfobutton integration with ClinGen clinical genomic resources CDSKB.org DocUBuild Use of ACMG genomic guideline ACT sheets to create genomic CDS Incorporation of CPIC Guidelines into ClinGen resource ClinGen Actionability Working Group

eMERGE, Electronic Medical Records and Genomics Network; ClinGen, Clinical Genome Resource; XML, Extensible Markup Language; EHR, electronic health record; HL7, Health Level 7; FHIR, Fast Healthcare Interoperability Resources; GA4GH, Global Alliance for Genomic Health; EHRI, Electronic Health Record Integration; CSER, Clinical Sequencing Exploratory Research; SMART, Substitutable Medical Applications, Reusable Technologies; ACMG, American College of Medical Genetics and Genomics; CPIC, Clinical Pharmacogenetics Implementation Consortium.

Several standards are required to implement the accurate rendering and scalable delivery of information to the clinician regarding genetic testing results. These involve the representation of patient genetic data; the representation of knowledge about genes, variants, and related phenotypes in a manner that can reflect knowledge updates; the robust definition of “genetic phenotypes”; the definition of interfaces to external knowledge resources; and the content and structure of information presented to the provider (**Table 1**).

### Storage of Genomic Data

Using genomic data in clinical practice will challenge the storage and computing capacity of current EHR systems. The potential volume of an entire genomic sequence, as opposed to a smaller number of genotypes, is beyond the capacity of current EHR systems. One solution to this problem is the ancillary genomic system (Starren et al., 2013). Much like an imaging archiving system, an ancillary genomic system can offer federated storage solutions optimized for the heterogeneity and size of genomic data and results. For example, an institution could receive from different laboratories a file containing star alleles for pharmacogenetic test results, an Extensible Markup Language (XML) file containing identified variants as part of a custom panel, or even a Variant Call Format (VCF) file for more expanded sequencing data, for the same patient. These data range in size from bytes to kilobytes to megabytes, respectively, and require distinct indexing approaches for fast retrieval. To leverage these data, an ancillary genomic system can perform specialized processing and be linked to the EHR to provide synthesized deeper views into genomic test results and associated data. Three eMERGE sites have developed and implemented versions of a genomic ancillary system. A prototype ancillary genomic system to support pharmacogenomic testing and reporting was implemented at Northwestern University (Rasmussen et al., 2019). Similarly, Mayo Clinic developed a genomic data warehouse (Horton et al., 2017). Partners HealthCare created a distributed system focused on managing indication-specific genetic testing (Aronson et al., 2012). However, open specifications for broadly targeted versions of such systems remain underdefined, and no open source solutions are currently available, although a few commercial systems have been developed to support pharmacogenomic data and single-gene or panel genetic testing. Ancillary genomic systems will be referenced in subsequent sections, emphasizing a key role in supporting the use of genomic information. Of note, EHR vendors are rapidly moving to cloud solutions to increase storage and accessibility of data while preserving EHR performance characteristics. These solutions have not yet been applied to genomic data, and there is concern that EHR vendors don’t understand the complexity of genomic data and haven’t been able to capture discrete results at the level of detail that is required in clinical care.

### Representation of Patient Genetic Data

Perhaps the most fundamental gap in all EHR implementations is the lack of a standardized, structured format for genetic data. Most of the data regarding genomic variants exists in the EHR as a scanned document stored in portable document format (PDF) (Shirts et al., 2015). Information in this form is static and does not provide an electronic point of reference to launch clinical information resources. Further, the naming conventions vary within and across institutions, so that tracking and monitoring results is difficult. To overcome the limited functionality of static documents, several healthcare systems have manually entered these results into data fields in the EHR, such as listing pharmacogenomic phenotypes as allergies or genetic findings as items on the problem list (Ohno-Machado et al., 2018). While these solutions are far from ideal, they do allow for CDS to be executed based on this information. However, as genomic results increase in number and complexity, *ad hoc* workarounds such as these become untenable due to the increasing amount of resources needed to maintain them and the risk for error inherent in any manual process. Up until this point, ancillary genomic systems, connected to the EHR, have been required to implement knowledge update–driven CDS (Aronson et al., 2012).

In addition to unstructured PDF reports, genomic test results also can be added to the EHR using Health Level 7 (HL7) Version 2 (v2) messages, which are widely supported across many clinical systems. The HL7 Clinical Genomics working group published an Implementation Guide to support the exchange of genomic data using v2 messaging and the Logical Observation Identifiers Names and Codes (LOINC) code system^4^. This approach enables the genomic results to be entered as structured data, which facilitates its use as part of a CDS system, but due to the message structure and the content available in LOINC, v2 messages are limited in their ability to render highly discrete genomic data components with semantic precision.

An emerging standard that has the potential to address some of these issues is the HL7 FHIR standard^5^. FHIR builds on prior HL7 standards but takes advantage of widely used web services technology, which facilitates implementer adoption. The HL7 v2 Clinical Genomic Report data structure does not map directly to the structures in the FHIR Genomics Reporting Implementation Guide. Harmonization of these two standards followed by implementation in laboratory information systems could accelerate the communication of genomic results between labs and clinics. A subgroup within HL7 Clinical Genomics is developing an information model for the clinical genomics domain, which is intended to provide common semantics for clinical genomics and serve as a harmonization point for genomic standards. Representatives from eMERGE and ClinGen are involved in this process and actively share lessons learned from site-specific implementation efforts.

In our experience through eMERGE, both the HL7 Clinical Genomic Report and the FHIR genomics standards have significant gaps, which hinders the adoption of these standards for clinical use. Given the heterogeneity of how genomic information is documented in the EHR, in preparation to establish a consensus format in eMERGE, the EHRI workgroup co-chairs designed and conducted informal interviews of eMERGE and Clinical Sequencing Exploratory Research (CSER) sites^6^ (Shirts et al., 2015). The goal of these interviews was to understand the intended use of genomic test reports and their requirements for transferring the reports and associated data from the laboratory to the sites. In summary, we found that sites wanted the reports in both a PDF and structured format, as well as the complete raw data files. Regarding transfer, secure file transfer protocol (SFTP) was available and acceptable to all sites; however, the ability to use a web service for transfer was not available at all sites. On the basis of these findings, the eMERGE Network created a consensus interface format to enable interorganizational transmission of genetic test results and genetic knowledge updates (available on GitHub)^7^. There is now an active effort within eMERGE to convert its existing XML format into a network-specific profile for genomic data.

Concerns exist about the risk of a privacy breach or discrimination based on the presence of genomic data in the EHR. This was the subject of a review article led by the EHRI and Ethical, Legal, and Social Issues working groups of eMERGE (Hazin et al., 2013). To date, there is limited evidence that this represents a significant problem, and additional protections specific to genetic data exist at the state and national level to protect against the inappropriate use of this information by health insurers and employers. As noted above, genetic information already is present in the EHR, albeit in a form less amenable to discovery. The incremental risk of providing the information in a more accessible form is offset by the improved ability of clinicians to use the information to improve patient care and outcomes. Therefore, this was not identified as a priority barrier by the respective working groups.

### Translating Variant Knowledge Into Genetic Phenotypes

**Figure 1:**
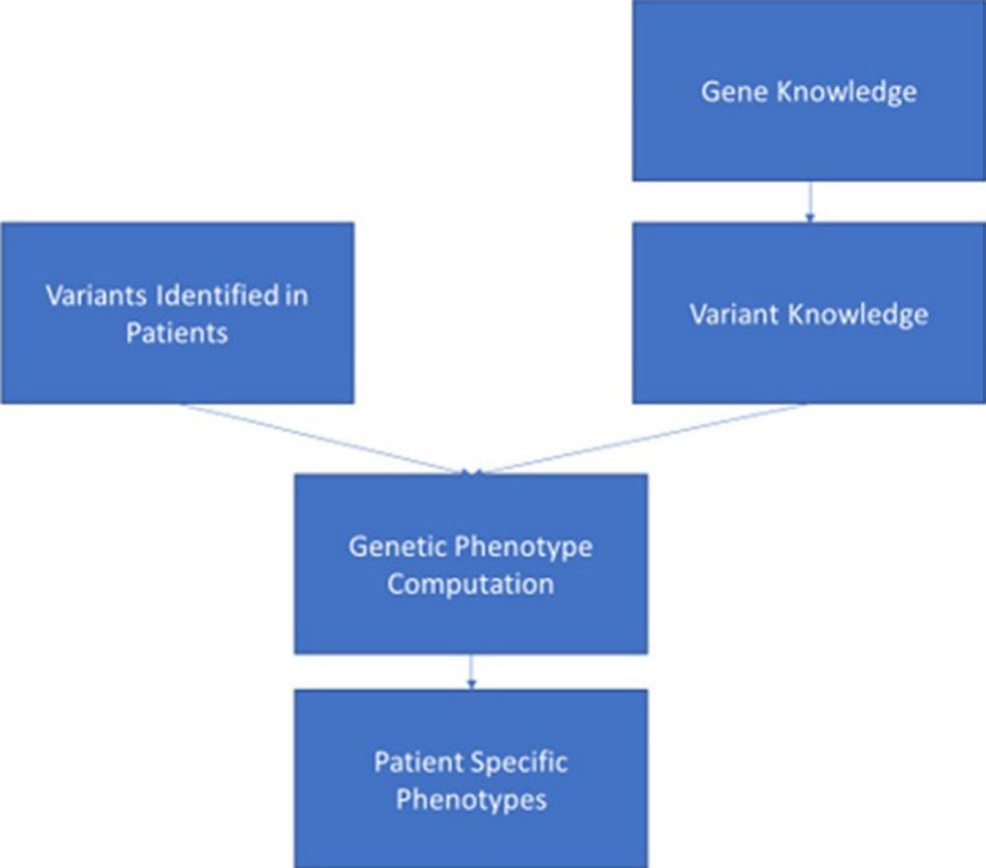
This figure depicts the ideal data flow for genomic variant data to be combined with knowledge associated with the gene and variant to generate a genetic phenotype that can be synthesized in the electronic health record to support clinician and patient decision making.

Assuming that genomic data can enter the EHR in a consistent, adequately structured electronic format, in order for the data to be used, it must be combined with standardized, computable genomic knowledge, which might exist at the variant, gene, and ultimately “genetic phenotype” levels. The genetic phenotype is a concept linking variant and gene knowledge to a defined patient characteristic or disease whose risk is associated with genetic variant(s) for which information can be delivered to clinicians (**Figure 1**). An example of such a phenotype is a patient with a pathogenic variant in the gene *BRCA1* [Online Mendelian Inheritance in Man (OMIM) gene #113705]^8^, which is associated with increased risk of developing breast, ovarian, and prostate cancers. The genetic phenotype associated with this pathogenic *BRCA1* variant is most commonly called “hereditary breast and ovarian cancer syndrome.” Such characteristics (such as the *BRCA1*-associated cancers) do not need to be present in the patient as the phenotype may consist of a risk or predisposition, as shown in **Table 2**. Precisely defining these genetic phenotypes requires more research. eMERGE and some ancillary genomic systems model the linkage between variants and diseases or pharmacogenomic effects. However, a more robust model that incorporates gene-level knowledge and relevant associated information (termed knowledge artifacts) is needed. A standard for these phenotypes is a necessary prerequisite as it serves as the launching point of genomic information resources. An early example of this is the Monarch initiative^9^ that is categorizing phenotypes from humans and other species to support discovery^10^. While not intended as a clinical resource, ClinGen has begun to incorporate some of the Monarch knowledge to support gene and variant annotation that ultimately yields information of relevance to clinicians.

**Table 2:** Examples of the relation between genomic variants and genetic phenotypes.

Type of result	Result	Genetic Phenotype	Description
Genetic disease diagnosis	Pathogenic variant *OTC* in a male	Ornithine transcarbamylase (OTC) deficiency	*OTC* is a gene on the X-chromosome, so a pathogenic variant found in a male would be expected to be associated with the disease OTC deficiency. It does not define the severity of the disease, which can range from hyperammonemic crisis in the newborn period to mild adult-onset forms. Note that sex must be specified, as the condition manifests differently in females.
Genetic predisposition	Pathogenic variant *BRCA1* in a female	Hereditary breast/ovarian cancer syndrome (HBOC)	A pathogenic variant in *BRCA1* results in increased risk for development of breast cancer (up to 80% lifetime risk) and ovarian cancer (up to 40% lifetime risk) in females. A male with a pathogenic variant would have an increased risk of breast cancer and prostate cancer.
Genetic carrier status	One ΔF508 variant in *CFTR*	Carrier for cystic fibrosis	Carrier status does not convey risk of disease for the individual but is relevant for reproductive decision making as there is increased risk of a child with CF if the partner is also a carrier.
Pharmacogenomic	*CYP2C19* *2/*2	Poor metabolizer	The presence of two variants that lead to decreased CYP2C19 enzyme activity affects the metabolism of drugs such as clopidogrel.

The granularity with which we standardize these genetic phenotypes and how that defines the focus of the information delivered is an important consideration. Precision medicine dictates that management is driven by a patient’s genetic variant results coupled with other relevant data. However, with millions of possible variants influencing human health and disease, the maintenance of information delivery at the variant level becomes a daunting task, as most diseases are driven by one of hundreds or thousands of different pathogenic variants in a gene. For example, *BRCA1* has more than 2,969 pathogenic variants asserted in the Clinical Variant Resource^11^ (ClinVar) as of May 2019. Genetic variant classification is done by laboratories as part of the result reporting process (Richards et al., 2015). The knowledge generated through this process can be captured in structured form. It can then be transmitted to the EHR ecosystem as structured results and, if necessary, revised as structured general “knowledge updates” when more is learned about a particular variant (Aronson et al., 2012). The eMERGE interface format supports transmission of knowledge updates related to these linkages. The ancillary genomic system approach has also been used clinically to manage these types of knowledge updates. Ideally, once this information reaches the EHR, it would then be combined with other genetic and non-genetic knowledge to determine patient genetic phenotypes. This last step is currently underdeveloped within EHR ecosystems.

The complexity of genetic disease underscores the importance of having a genetic phenotype as a point of decision making and information delivery in the EHR. There are cases such as with alpha-1-antitrypsin deficiency for which specific variants are associated with variable severity, and environmental factors such as smoking dramatically alter the risk of developing chronic obstructive pulmonary disease and, by necessity, alter the recommended care (Al Ashry and Strange, 2017). It is unrealistic to expect that clinicians will wade through pages of documentation to discover the specific risks associated with that variant. Thus, having the most pertinent information delivered according to the relevant combination of variants and clinical variables is a key goal of CDS. This problem will increase exponentially as we apply genetic variation and non-genetic modifiers to each patient. More effort to increase the granularity of genetic phenotypes may save substantial time and effort on the part of the clinician in the long run, as well as provide better care.

The lack of standardized terminologies for genetic phenotypes for use in result reporting can lead to clinician confusion, while also impacting interoperability and implementation of CDS. Consider the genetic phenotype “hereditary breast and ovarian cancer syndrome.” While this term is in common use, the lack of a standard terminology could result in one lab reporting the genetic phenotype as “*BRCA1*- and *BRCA2*-associated hereditary breast cancer,” while another may report it as “breast–ovarian cancer, familial 1.” In the former case, a clinician unfamiliar with the gene–disease association may only provide information about breast cancer, which is not consistent with evidence-based recommendations. This was a significant issue in pharmacogenomics for which use of different terms (extensive metabolizer, normal metabolizer) for the same pharmacogenetically defined phenotype led to confusion (Caudle et al., 2017). Assignment of these variants to the correct phenotype is critical, as the phenotype is the data element to which all information resources are mapped, and it is a key criterion for CDS interventions. Without standardization, the healthcare system must resort to either manual assignment of the phenotype or mapping of phenotypes for each laboratory they use and for every condition for which the laboratory tests. In recognition of this issue, the Clinical Pharmacogenetics Implementation Consortium (CPIC) led an effort to harmonize terms for reporting that incorporated the input of non-specialist clinicians to develop a standard terminology for reporting that is consistent and unambiguous, thus enhancing clinician understanding. A related effort to harmonize terms describing phenotypes and outcomes involving the eMERGE Outcomes working group, and the ClinGen Actionability Working Group (Williams et al., 2018) provides a basis for work by informaticists to create terminology standards to enhance interoperability. ClinVar and ClinGen as public repositories could play a decisive role in managing the known associations between variants and genes, and the resulting genetic phenotypes.

### Clinical Decision Support for Clinical Genomics

It is not logical nor feasible for EHR vendors and most healthcare systems to create and maintain large-scale genomic knowledge resources for clinicians. This reality necessitates the ability of the EHR to access external knowledge content and CDS capabilities, ideally through scalable standards-based approaches as proposed by Welch et al. (2014) and Shellum et al. (2016). Our previous summary of opportunities for genomic CDS illustrates that there is much we can learn from implementing CDS in the pre-genomic era (Overby et al., 2013). CDS can be organized into three general categories: passive, asynchronous (or semi-active), and active (Lobach et al., 2012). Passive CDS provides just-in-time access to information resources triggered by the clinician when a clinical question is raised. Asynchronous CDS presents aggregated information to a clinician to support patient-specific care reassessments based on new knowledge, or as part of quality improvement and care initiatives for a group of patients outside of an individual patient encounter. Based on EHR user events (e.g., chart opening, medication prescription, laboratory results review), active CDS provides information to clinicians in real time at the point of care specific to the patient encounter anticipating that clinicians will not always be aware that information is needed to make a clinical decision.

Several CDS modalities, such as alerts and reminders (active or asynchronous CDS), population health management dashboards (asynchronous CDS), infobuttons (passive CDS), and integrated information displays (active, asynchronous, or passive) can be used to help providers integrate clinical genomics into routine patient care decisions. For example, *alerts* can prompt providers when a patient may benefit from a certain pharmacogenomic test or when the result of a test warrants changes in the patient’s medication or management (active CDS) (Herr et al., 2019). *Reminders* (active or asynchronous CDS) serve as a checklist to help providers follow various evidence-based preventive measures, including cancer screening approaches (such as an accelerated schedule for routine colonoscopies in a patient with a genetic predisposition to developing colorectal cancer) that are personalized based on clinical genomics (Aronson et al., 2012). Patient-specific knowledge alerts (asynchronous CDS) can alert clinicians outside of an encounter when new information emerges on a variant previously identified in a patient. *Population health management* (asynchronous CDS) uses a different approach, whereby patient records are automatically scanned to identify and aggregate those who meet criteria for certain genetic evaluation or care based on a previously reported genetic result (Kohlmann et al., 2019). *Infobuttons* (passive CDS) provide just-in-time access to external knowledge resources accessible by but not necessarily contained within the EHR. Based on the context of the interaction between the provider and the EHR, infobuttons (Cook et al., 2017) are found next to items in different sections of the EHR, such as problem list, medications, orders, and laboratory test results. Infobuttons have been a key strategy to present genetic information to clinicians as part of both the eMERGE and ClinGen and will be discussed below (Overby et al., 2014; Heale et al., 2016; Crump et al., 2018). Complementary technologies for passive CDS are being developed that enable the delivery of genomic results *via* mobile devices (Samwald and Freimuth, 2013). *Integrated information displays* provide intelligent visualization of patient data integrating multiple sources within and outside the EHR and can be used to display genetic data along with other relevant clinical data in the EHR.

**Figure 2:**
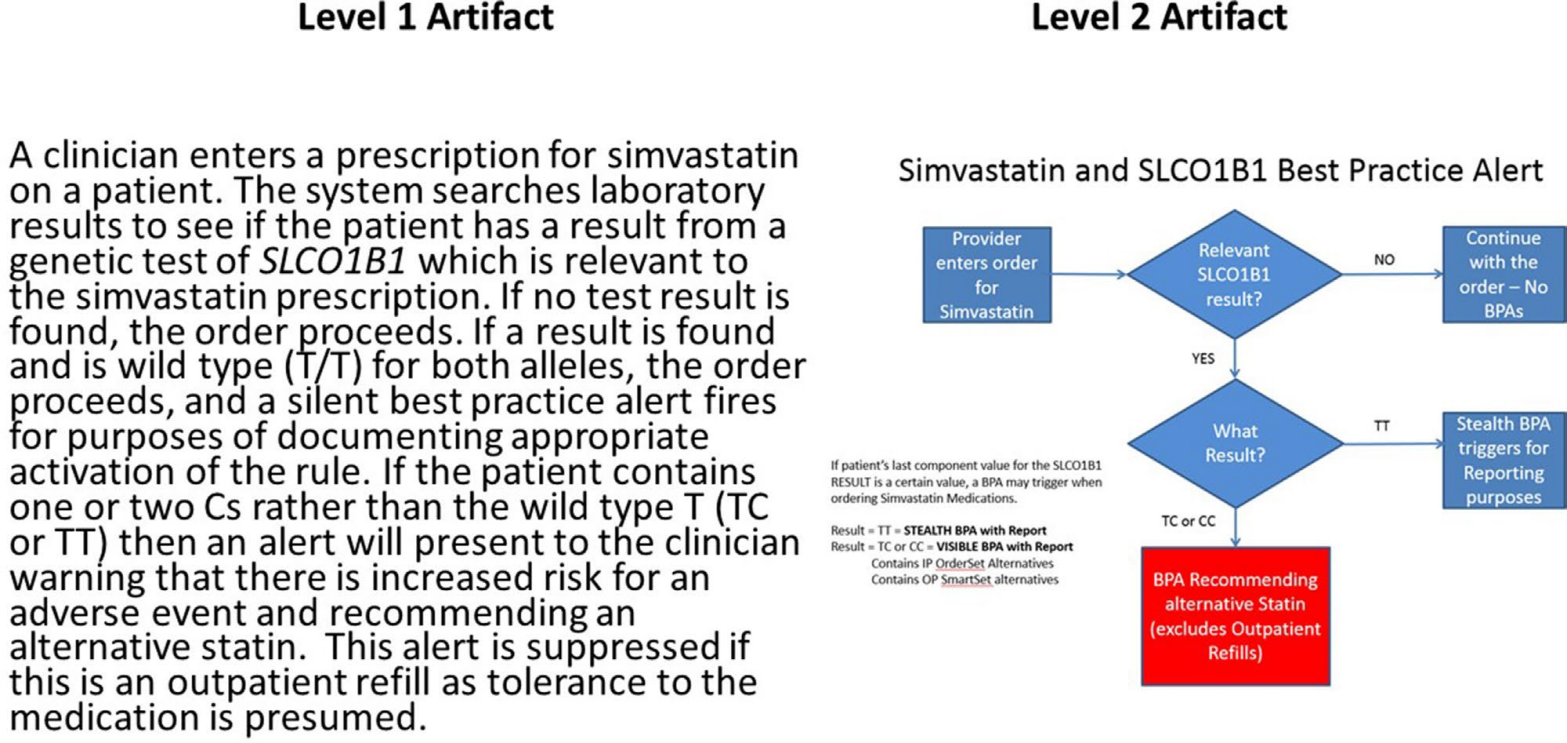
Example of narrative or L1 (left) and wire frame or L2 (right) clinical decision support artifacts for a pharmacogenomic use case involving the simvastatin:*SLCO1B1* drug:gene pair. Presence of the *5 allele in one or both copies of *SLCO1B1* is associated with an increased risk of adverse events involving inflammation of the muscle (myositis). Of note is decision logic that suppresses the alert if the patient is already on the medication as this implies the absence of the adverse event related to the exposure. This reduces disruption of the clinician workflow. This artifact and many other examples are available at CDSKB.org. Free registration is required.

While critical to help providers integrate clinical genomics in routine patient care, several challenges limit the implementation and adoption of CDS for clinical genomics. Overall, any basic CDS requires access to EHR data in a standard, structured, and computable format. However, as mentioned above, the absence of relevant vocabulary and messaging standards is a critical barrier. Even where these exist, there is low adoption of standard vocabularies for genetic tests and standards for the representation of genetic test reports in a computable format. Similarly, although standard representations of CDS logic have existed for decades (most notably Arden syntax (Hripcsak et al., 2018)), these have not seen widespread adoption in commercial EHRs. This means that institutions wishing to disseminate successful CDS implementations need to do so either using what the CDS Consortium (Middleton 2009) has termed Level 1 artifacts (Hongsermeier et al., 2011)—that is, narrative descriptions of CDS logic—or by distributing entire applications that implement the CDS, few of which exist for genomics. In an attempt to capture Level 1 artifacts, the eMERGE Network in conjunction with the NHGRI-funded Implementing Genomics in Practice (IGNITE) consortium developed the CDS Knowledge Base^12^ (CDSKB), which includes a dedicated library for the dissemination of genomic CDS. While primarily populated with Level 1 and Level 2 (flowcharts or wire frame) artifacts, as shown in **Figure 2**, it is capable of storing computable definitions (Levels 3 and 4), renderings of which have been explored for pharmacogenomics (Linan et al., 2015). To better understand the complexities of genomic CDS, eMERGE Network sites examined issues related to CDS implementation using pharmacogenomics as the use case (Herr et al., 2015). However, given the complexity of CDS logic in clinical genomics, it would be desirable for EHR systems to defer clinical genomic CDS to external web services. While not universal, the rapid adoption of emerging CDS standards such as CDS Hooks^13^, which takes advantage of Application Programming Interfaces (APIs) in the EHR, has the potential to enable a cloud-based ecosystem for clinical genomics as demonstrated in recent prototype work in pharmacogenomics (Dolin et al., 2018).

### Infobuttons

Infobuttons are particularly appealing for clinical genomics because they are required for EHR certification in the United States Meaningful Use program (Federal Register, 2012); leverage external genomic resources; and can provide just-in-time access to relevant and up-to-date clinical genomics information under the clinician’s control. This approach, which mapped infobuttons to existing publicly available resources, was successfully implemented in a non-commercial EHR system in 2006 (Del Fiol et al., 2006), well before the HL7 Infobutton Standard was developed. Once included in EHR certification, both eMERGE and ClinGen have studied the use of infobuttons for delivery of genomic knowledge at the point of care, as there are no other generalizable solutions in commercially available EHR systems.

There are barriers besides those discussed above that hinder the implementation of infobuttons for clinical genomics. First, with the exception of the Pharmacogenetic Knowledgebase ^14^(PharmGKB), clinical genomic resources are not compliant with the HL7 Infobutton Standard (Del Fiol et al., 2012; Strasberg et al., 2013), which is the mechanism used by EHR systems to communicate with external knowledge resources (Heale et al., 2016). Second, not all clinical genomic resources provide access to actionable recommendations in a format that can be readily accessed at the point of care. Last, EHR systems are unable to distinguish the context in which a clinical genomics resource might be useful, requiring the use of external web services such as OpenInfobutton (Del Fiol et al., 2013). The eMERGE and ClinGen EHR working groups have been working cooperatively to overcome these barriers.

Efforts are underway through the eMERGE Network and ClinGen to develop infobutton-compliant genomic resources to deliver targeted information to patients and providers (Overby et al., 2014). A survey of eMERGE and CSER consortia sites identified that existing resources contain the content that an institution would like to present at the point of care but may require some additional synthesis (selecting particular sections or paragraphs), localization (providing institution-specific information such as the contact information for genetic counselor referrals), and branding (institution logos for patient handouts) (Rasmussen et al., 2016). In addition, the adoption of a structured template would also benefit content authors to ensure that resources sufficiently answer anticipated questions for genomic medicine (Overby et al., 2014). More recently, the eMERGE Network has led the development of a tool called DocUBuild^15,16^, which is a freely accessible and open source platform to create information resources to support genomic medicine. DocUBuild supports features such as templating, content sharing, and localization (with linked provenance), as well as branding. While still in its infancy, DocUBuild is providing a testing ground to evaluate how genomic resources may be better optimized for patients and providers.

eMERGE and ClinGen are also working collaboratively with the ACMG on the ACT sheets (ACMG, 2001)^17^. ACT sheets were initially developed to support clinician information needs related to newborn screening programs. They were designed to be used as point-of-care educational documents that provide clinicians with sufficient knowledge about a rare genetic condition they had likely not encountered previously and included recommendations on care needed to optimize patient outcomes. They were designed to include both a narrative summary (L1) and decision tree (L2) CDS artifacts. As genetic and genomic indications expanded, the content of the ACT sheets has extended to cover more indications. In particular, ACT sheets are under development to support the care of patients receiving a result from the ACMG secondary findings list (Kalia et al., 2017). The goal of this collaboration is to use these ACT sheets to develop computable CDS that can be distributed through EHR systems, lowering the burden of implementation for systems implementing genomic information into clinical care.

### Integrated Information Displays *Via* EHR Apps

An increasingly popular approach to integrating CDS capabilities into EHR systems is the Substitutable Medical Applications, Reusable Technologies (SMART) coupled with FHIR (SMART on FHIR) (Mandel et al., 2016). SMART enables applications to be integrated for interoperability across different EHR vendors, including single sign-on, end point for users to launch an app from within the EHR, and exchange of security token for apps to access the EHR’s FHIR server. Examples of SMART on FHIR apps with integrated information displays for clinical genomics are available (Alterovitz et al., 2015; Warner et al., 2016). In addition to including general patient genetic test result management functionality, apps such as these could be used to provide deep disease-specific functionality that combines genomics with other forms of relevant clinical data. Other solutions are also being explored. Partners HealthCare implemented an EHR integrated app, before the advent of the SMART on FHIR standard, to manage genetic results and associated knowledge (Aronson et al., 2012).

Building, clinically validating, integrating, and distributing these apps is resource intensive. In part, this is due to the complex nature of genomic data and the knowledge required to process the results into clinically actionable interpretations. Although many resources exist that contain this knowledge (e.g., CPIC Guidelines^18^, ACMG ACT sheets^17^), not all are currently available in a computable form. This is an additional challenge to the ones listed above regarding the representation of such knowledge. There are other issues with clinician adherence to guidelines that are not specific to genomics but must be recognized if guideline-based care is to be realized. Examples include inclusion of language that is not adequately explicit and therefore difficult to compute (e.g. “might consider” or “1 to 2 years”); the source of the guideline; differences in clinical workflow; clinician knowledge; and differences in management approaches by different specialties, among others (Cabana et al., 1999).

Having the data represented in a computable form will allow developers to more easily integrate these sources of information, reducing development time and duplication of the knowledge bases, as well as facilitating more rapid updates as knowledge changes. In addition, standards such as SMART and FHIR are not implemented equally across all EHR vendors and even across instances of the same vendor’s EHR. As these standards continue to see adoption and maturation, ongoing validation and communication with vendors is needed to ensure that the implementations are delivering on the promise of the technology.

### Access to Genomic Knowledge

ClinGen’s website, www.clinicalgenome.org, was established to support ClinGen’s mission to “provide high quality, curated information on clinically relevant genes and variants” (Rehm et al., 2015) in a centralized way to the public. ClinGen’s website was launched in 2014, and over the last 5 years, the website has undergone many improvements to enhance the ability to connect curations to the genomics community and the EHR.

In 2015, ClinGen provided access to ClinGen’s curations and external genomic resources by releasing an infobutton-enabled search interface built into a section of the website. This update enabled ClinGen’s website to utilize the HL7 Infobutton Standard (Del Fiol et al., 2012) to allow visitors to query a term related to other standard nomenclatures [OMIM, Human Genome Organization (HUGO) Human Gene Nomenclature Committee (HGNC), RxNorm] and have information from a variety of genomics resources to be presented to the user through the use of web standards and external links to resources.

Throughout 2016 and 2017, ClinGen improved the ability to query terms (OMIM, Orphanet, HGNC, RxNorm) and moved the search feature to ClinGen’s home page. At this time, updates were made to allow ClinGen’s website to support basic HL7 Infobutton-compliant requests and display curation knowledge generated by ClinGen’s curation groups. In 2018 and 2019, ClinGen continued to make improvements by including support for multiple disease resources through the use of the Monarch Disease Ontology (MONDO), allowing ClinGen’s curations to be directly published to the website from the curation interfaces after approval, and by investing resources to expand the depth of the curation knowledge available to the public.

As of June 3, 2019, ClinGen’s website provided curated information on 747 Gene–Disease Clinical Validity Summary Curations, 102 Clinical Actionability Curations, and 1,475 Dosage Sensitivity Curations. ClinGen’s Evidence Repository provides information about 684 Variant Pathogenicity Curations.

We have learned that the technical process for a website to implement basic support to become HL7 Infobutton compliant is straightforward and relatively easy to get started. The process to go further by providing a web resource that fully utilizes HL7 Infobutton and/or supports SMART on FHIR requires a commitment of resources and assessment to understand specific use cases. Resources should consider how their tools may be adopted and utilized within the EHR. This is an endeavor that each resource should undertake wisely, and resources should consider conducting usability studies to assess the user experience of the resource within the EHR.

Over the last 4 years, we have successfully been able to display ClinGen’s curations and provide access to external genomics resources through the use of OpenInfobutton (Heale et al., 2016) by making our resource HL7 Infobutton compliant. We are continually working to improve the resource and information we offer, explain how genomic resources can become infobutton compliant, and promote the infobutton adoption in EHR platforms. Recognizing that infobuttons are not routinely “turned on” in most healthcare organizations, the ClinGen EHR WG has developed an implementation guide specific for OpenInfobutton access to the ClinGen resource that is freely available^19^.

## Discussion

Integration of structured genomic information into the EHR to support patient care remains limited. Ongoing work at the national and international level is targeting the barriers described above. The HL7 FHIR specification is under active, collaborative development by a wide variety of stakeholders, including national initiatives. In particular, the Office of the National Coordinator’s (ONC’s) Sync for Genes^20^ precision medicine research program recently sponsored the pilot implementation of the FHIR Genomics specification, which will be used by the *All of Us* Precision Medicine Initiative. In another international effort to develop standards for genomics, the Global Alliance for Genomic Health (GA4GH)^21^ is developing a suite of tools and specifications that enable genomic data sharing. The GA4GH is informed by FHIR but does not utilize FHIR. Representatives of eMERGE and ClinGen are working with HL7 and GA4GH leadership to keep the two projects aligned to reduce the risk of development of different standards that are incompatible. The standards developed by HL7 and GA4GH will require substantive changes in as well as enhancement to the currently available vendor-based EHR, laboratory, and ancillary genomic systems to achieve full integration. Projects focused on implementation of genomics in clinical care, such as eMERGE and ClinGen, provide a valuable test bed for the development, testing, optimization, and dissemination of best practices.

To date, most of the research has been focused on feasibility with relatively limited network-wide implementation. Future efforts must focus on the end user to measure the effectiveness of these modalities for education and support of clinicians and patients, and ultimately on the impact of genomic medicine. An early example of this is focused on the implementation of pharmacogenomics in eMERGE Phase 2 (Rohrer Vitek et al., 2017). The 10 sites implementing pharmacogenomics catalogued their strategies for clinician education. While not focused on the effectiveness of the educational interventions, this survey collects a broad range of approaches providing the basis for comparative testing of the effectiveness of the strategies. Another working group of ClinGen, Consent & Disclosure Recommendations (CADRe), is beginning to study this issue. CADRe has developed recommendations regarding consent and results disclosure for genomics focused on clinicians without training in genetics (Ormond et al., 2019). They are now working to develop educational materials to support the integration of CADRe recommendations into practice at the point of care. CADRe has initiated engagement with clinicians to guide development of the educational strategies, which will ultimately be included as part of the ClinGen resource.

Representatives of eMERGE and ClinGen are actively participating in various international standards development efforts, including those in HL7 (FHIR genomics) and GA4GH. The practical experience from early genomic medicine implementation efforts is critical to test the usefulness of existing and proposed standards. An example of this is the selection of ClinGen as a driver project for the GA4GH. The specific project is focused on the development of standards for data sharing (Dolman et al., 2018). These collaborations will accelerate the development and testing of standards necessary to overcome the barriers identified above.

One other consideration is the sustainability of the current efforts. eMERGE and ClinGen are funded research projects, raising the question of how such efforts can be sustained over time. This is particularly critical for the ClinGen resource, which is increasingly viewed as a foundational genomic knowledge resource essential for the clinical use of genomic information. Transition of the resource from a research project to some other sustainable model is essential, and discussion of alternative models has begun. Recognition of the value of the resource by a diverse set of stakeholders is essential to ensure investment and innovation to support sustainability.

In conclusion, eMERGE and ClinGen in conjunction with many other efforts in the US and internationally are working to develop educational approaches within the EHR to support clinicians to integrate genomic information in clinical care. While much work remains, the lessons learned from these projects have provided rich information that can be used to advance the field. Efforts to engage with clinicians as end users to understand preferences and measure effectiveness are needed.

## Executive Summary

- For genomic medicine and precision health to improve patient outcomes, credible information must be available to clinicians in time to support clinical decision making.
- The electronic health record (EHR) is a tool that can provide genomic information and associated knowledge to clinicians at the point of care.
- Barriers to the use of EHRs for genomics have been identified, and potential solutions are emerging (see **Table 1** for details). These include:
  - Lack of standards to represent and communicate genomic information.
  - Inability to store genomic information in current EHR systems.
  - Translating genomic variants into clinical phenotypes that clinicians can recognize and use to manage patients.
  - Access to reliable genomic knowledge sources.
  - Existing efforts are largely supported by institutional and grant funding. Sustainable models are needed for further development.
- EHR systems have some capabilities that can be used to overcome some of the barriers, but the solutions are not generalizable at present. Examples include:
  - Clinical decision support systems that can be modified to support some genomic medicine interventions.
  - Infobuttons (context-sensitive information retrieval tools) linked to genomic information resources.
- Resources of genomic knowledge such as the Clinical Genome Resource (ClinGen) are developing and are being made accessible to tools within the EHR, lowering barriers for use in a clinical setting.
- eMERGE and ClinGen in conjunction with many other efforts in the United States and internationally are working to develop educational approaches within the EHR to support clinicians to integrate genomic information in clinical care. Lessons learned from these projects have provided rich information that can be used to advance the field.

## Data Availability Statement

The datasets generated for this study are available on request to the corresponding author.

## Author Contributions

Conception of manuscript: MW. Conduct of research: MW, NW, SG, RF, LR, CT, SA, GF, EH, and GW. Writing: MW, NW, SG, RF, LR, CT, SA, and GF. Critical review of manuscript: MW, NW, SG, RF, LR, CT, SA, GF, CW, WC, GJ, JN, GW, EH, AF, CP, and RR.

## Funding

Brigham and Women’s Hospital: U01HG8685 (eMERGE); Cincinnati Children’s Hospital Medical Center: U01HG8666 (eMERGE); Columbia University: U01HG008680 (eMERGE); Geisinger Clinic: U01HG8679 (eMERGE) and U41HG009650 (ClinGen); Group Health Cooperative/University of Washington: U01HG8657 (eMERGE); Mayo Clinic: U01HG063798 (eMERGE) and U41HG068345 (ClinGen); Northwestern University: U01HG8673 (eMERGE); Partners HealthCare/Broad Institute: U01HG8676 (eMERGE); Vanderbilt University Medical Center: U01HG8672 (eMERGE).

## Conflict of Interest

SA: Partners HealthCare receives royalties from sales of GeneInsight software. Group has received funding from Persistent Systems and Novartis to construct SMART on FHIR applications and infrastructure. The remaining authors declare that the research was conducted in the absence of any commercial or financial relationships that could be construed as a potential conflict of interest.

The reviewer YB declared a past co-authorship with one of the authors GPJ to the handling editor.

## Footnotes

1
https://www.genome.gov/Funded-Programs-Projects/Electronic-Medical-Records-and-Genomics-Network-eMERGE.

2
https://emerge.mc.vanderbilt.edu/the-emergeseq-platform/.

3
https://emerge.mc.vanderbilt.edu/projects-2/ehr-integration/.

4
https://www.hl7.org/implement/standards/product_brief.cfm?product_id=23.

5
https://www.hl7.org/implement/standards/product_brief.cfm?product_id=343.

6
https://cser-consortium.org/.

7
https://github.com/emerge-ehri/results-schema.

8
https://omim.org/entry/113705?search=113705&highlight=113705.

9
https://monarchinitiative.org/.

10
https://monarchinitiative.org/page/about.

11
https://www.ncbi.nlm.nih.gov/clinvar/.

12
https://cdskb.org/.

13
https://cds-hooks.org/.

14
https://www.pharmgkb.org/.

15
https://docubuild.fsm.northwestern.edu/

16
https://www.genomeweb.com/informatics/researchers-develop-web-app-improve-curation-delivery-genomic-knowledge-point-care

17
https://www.ncbi.nlm.nih.gov/books/NBK55832/

18
https://cpicpgx.org/guidelines/

19
http://www.openinfobutton.org/documentation

20
https://www.healthit.gov/sites/default/files/sync_for_genes_report_november_2017.pdf

21
https://www.ga4gh.org/
